# Epcoritamab plus R² in follicular lymphoma: practice-changing efficacy meets unresolved toxicity trade-offs

**DOI:** 10.3389/fonc.2026.1811781

**Published:** 2026-03-24

**Authors:** Qinghua Ke, Shiqiong Zhou

**Affiliations:** Department of Chemoradiotherapy, The First People’s Hospital of Jingzhou, Jingzhou, Hubei, China

**Keywords:** bispecific antibody, epcoritamab, follicular lymphoma, infection risk, R² regimen, toxicity

## Introduction

The EPCORE FL-1 trial demonstrates that adding epcoritamab to lenalidomide and rituximab (R²) significantly improves progression-free survival and complete response rates in patients with relapsed or refractory follicular lymphoma, with a 79% reduction in the risk of disease progression or death. However, this efficacy comes at the cost of substantially increased grade ≥3 adverse events, particularly infections, raising concerns about toxicity management, patient selection, and real-world applicability. While the regimen offers a promising chemotherapy-free option, unresolved questions regarding long-term survival, tolerability in older or comorbid populations, and access in resource-limited settings remain. Further research is needed to refine patient selection and optimize supportive care.

The EPCORE FL-1 trial, published in The Lancet, establishes fixed-duration epcoritamab combined with lenalidomide and rituximab (R²) as superior to R² alone in relapsed or refractory follicular lymphoma, achieving a 79% reduction in the risk of disease progression or death (hazard ratio 0·21, p<0·0001) (1). As the first phase 3 study to report on a bispecific antibody combination in this setting, these results position epcoritamab plus R² as a potential new standard for second-line or subsequent therapy. However, while the efficacy signal is undeniable-–complete response rate nearly doubling from 50% to 83%-–the substantial increase in grade ≥3 adverse events (90% vs 68%) and infectious complications raises critical questions about patient selection, toxicity management, and real-world generalisability that warrant closer examination.

## A new benchmark for chemotherapy-free combinations

The magnitude of benefit observed with epcoritamab plus R² is remarkable by any measure. A 79% risk reduction in progression-free survival, with 16-month estimates of 85·5% versus 40·2%, represents a genuine leap forward for a population where R² alone yielded median progression-free survival of only 11·7 months in this trial. The complete response rate of 83%-–consistent across high-risk subgroups including POD24 (progression of disease within 24 months) and double-refractory disease-–suggests that adding a CD3×CD20 bispecific antibody effectively deepens responses beyond what immunomodulation and CD20 targeting alone can achieve.

The mechanistic rationale is sound: epcoritamab binds a unique CD20 epitope distinct from rituximab, enabling simultaneous T-cell engagement without competitive interference (2). Preclinical data demonstrating enhanced natural killer cell activation and rituximab-mediated cytotoxicity provide biological plausibility for the triplet’s synergy (3). From a clinical practice standpoint, the fixed-duration, outpatient subcutaneous administration with step-up dosing to mitigate cytokine release syndrome (CRS) makes this regimen feasible across diverse healthcare settings, provided appropriate monitoring infrastructure exists.

## The toxicity ceiling: infection risk as the unresolved variable

Despite these efficacy gains, the safety profile demands sober assessment. Grade ≥3 infections occurred in 33% of epcoritamab-R² recipients versus 15% with R² alone, with opportunistic infections-–particularly cytomegalovirus and herpes virus-–observed in 18% versus 4%. While antimicrobial prophylaxis was implemented (87% received Pneumocystis jirovecii prophylaxis, 71% herpes virus prophylaxis), the 6% rate of febrile neutropenia and median neutropenia duration of 22 days indicate that prolonged cytopenias and immune compromise are inherent to this triplet.

The critical unanswered question is whether these toxicities will prove manageable in real-world populations less selected —in terms of general health status variables— than trial participants. EPCORE FL-1 enrolled patients with median age 60–63 years, good performance status, and excluded those with significant comorbidities. Community oncology practices treating older adults with cardiovascular disease, renal impairment, or baseline cytopenias may encounter higher rates of treatment discontinuation or infectious mortality than reported here. Notably, 19% of epcoritamab-R² recipients discontinued treatment due to adverse events-–primarily infections-–raising concerns about whether less resilient patients can complete the intended 12-cycle course. Moreover, tolerability of the triplet may negatively affect relative dose intensity (RDI). The relationship between RDI of the triplet and its components and long-term efficacy outcomes (PFS, OS) should be evaluated during long-term follow-up. Despite subsequent salvage therapies, such analyses could yield valuable data to outline a baseline patient profile foreboding likely inadequate treatment tolerance.

## Open-label design and immature survival data

The open-label design, while acknowledging the obvious advantages in this context—simplifying logistics, enabling safe and timely management of immune toxicities, and avoiding placebo procedures—while still maintaining scientific rigor when objective endpoints are used, introduces potential bias in adverse event reporting and subsequent therapy decisions. Although independent review committee assessment of primary endpoints mitigates efficacy bias, toxicity assessments remain vulnerable. More importantly, overall survival data are early and preliminary, with only 24% of required events observed. Properly pre-planned long-term follow-up evaluations in clinical studies and real-world evidence generation are essential in indolent lymphomas. The current hazard ratio of 0·38 (95% CI 0·18--0·80) favours epcoritamab-R², but the 16-month survival estimate of 95·8% versus 88·8% reflects early separation driven largely by fewer progression-related deaths. Whether this translates into durable survival advantage or merely delays progression without altering ultimate outcomes-–given effective salvage options including CAR T-cell therapy-–remains to be established. Long-term follow-up and/or real-world evidence generation may help elucidate whether efficacy of subsequent CAR-T in follicular lymphoma is not negatively affected by previous bispecific antibody treatment, similar to what Crochet et al. revealed in DLBCL (4).

Furthermore, the control arm’s progression-free survival of 11·7 months is notably shorter than the 39·4 months observed in the AUGMENT trial (5). This discrepancy reflects inclusion of higher-risk patients in EPCORE FL-1-–all required GELF criteria for active therapy, and 41% had POD24. It is critical to emphasise that AUGMENT excluded rituximab-refractory patients and did not mandate treatment by GELF criteria. The EPCORE FL-1 population, comprising patients with prior immunochemotherapy and high rates of POD24 and double-refractory disease, represents a significantly higher-risk group. This context is essential to prevent readers from undervaluing R² in lower-risk relapsed settings. Comparisons with the recently reported inMIND trial, where tafasitamab plus R² achieved a 57% risk reduction with lower complete response rates (45%) but also lower grade ≥3 infections (18%), highlight divergent benefit-risk profiles across CD19-directed and CD20-directed combinations (6).

## Subgroup nuances and unanswered questions

While subgroup analyses uniformly favoured epcoritamab-R², sample size limitations preclude definitive conclusions about patients with recent bendamustine exposure-–only seven participants received bendamustine within 12 months. Given evidence that bendamustine induces T-cell exhaustion and impairs response to T-cell engaging therapies in DLBCL (7), far less clear evidence exists for negative interference with bendamustine pretreatment in follicular lymphoma than in DLBCL. In FL, only CD4 counts are decreased with no obvious efficacy loss (8). In such cases, the risk-benefit ratio must be carefully weighed, and alternative mechanisms of action-–where supported by data and available-–may be considered, although tafasitamab-based regimens or CAR T-cell therapy may also be influenced by bendamustine-induced T-cell dysfunction.

Additionally, the issue of grade 3B follicular lymphoma is highly relevant in another sense. While 3B FL patients are generally considered eligible for DLBCL regimens per guidelines and are typically excluded from trials like EPCORE FL-1, occult transformation can be unexpectedly high (76% in the Freeman retrospective analysis) (9). A potential tissue sample reanalysis in the POD24 subgroup would be meaningful—though likely not feasible—with a re-evaluation of transformed FL versus true POD24 efficacy correlates. Whether epcoritamab-R² can prevent or delay transformation events remains unknown, as event-free survival data were immature at analysis.

## Global access and health system implications

From a global oncology equity perspective, epcoritamab-R² presents challenges. Subcutaneous administration facilitates outpatient delivery, but requirements for CRS monitoring, infection prophylaxis, and management of prolonged cytopenias demand healthcare infrastructure not universally available. In low-resource settings where R² alone may already be inaccessible, adding a costly bispecific antibody risks widening disparities. Cost-effectiveness analyses will be essential to guide reimbursement decisions and prioritisation within national formularies. With upcoming regulatory HTA assessments, future EPCORE FL-1 cost-effectiveness models versus R², mosunetuzumab, tafasitamab-R², and CAR-T will commence.

The observation that quality of life (FACT-Lym scores) was preserved during treatment provides some reassurance, but these data derive from highly selected trial participants with high baseline scores. Mean change analyses can mask significant deterioration experienced by patients who suffer serious adverse events, such as hospitalisations for infections. Future analyses should report the proportion of patients experiencing clinically meaningful deterioration, not merely average scores, to better capture the tolerability landscape. Thus, while the regimen did not lead to net QoL deterioration at the population level-–likely due to the benefit of disease control-–this finding should not be interpreted as uniform tolerability across all patients.

## Redefining the second-line landscape

Where does epcoritamab-R² fit among emerging options? Compared with tafasitamab-R², it offers deeper responses but higher toxicity. Compared with CAR T-cell therapy-–curative-intent but logistically complex and associated with unique toxicities-–epcoritamab-R² provides an immediately available, fixed-duration alternative. The choice may ultimately hinge on individual patient risk profiles: younger, fitter patients might prioritise depth of response and accept higher toxicity, while older or frailer individuals might prefer regimens with lower infection risk even at the cost of lower complete response rates. As the haunting ‘ideal’ patient profile for epcoritamab-R² shapes a young, high-performance status POD24 patient, and at least indirect comparison with autologous hematopoietic stem cell transplantation deployed in second-line FL is tempting (10).

## Future directions

The EPCORE FL-1 investigators deserve credit for delivering practice-changing results. However, several priorities emerge. First, longer follow-up is essential to establish whether complete responses translate into durable remissions or prolonged treatment-free survival, rather than implying cure in a disease not generally considered curable in the relapsed setting. Second, with the advent of new options with novel mechanisms of action, a new set of adverse events must be addressed. With relevant scientific bodies issuing specific guidelines to address these, treating physicians may be empowered to exploit the full potential of advanced, often chemo-free modalities. In this specific case, recommendations such as the one issued by the British Society for Haematology (BSH) (11) can help alleviate the infectious side-effect burden of the practice-changing epcoritamab-R² treatment in second-line FL. These recommendations also encompass antifungal prophylaxis and, crucially, necessary immunoglobulin replacement measures—the latter typically not mandated in clinical studies for various understandable reasons, but omission may endanger the positive outcome of any drug evaluations in B-cell malignancies. Third, translational analyses should identify biomarkers predicting infection risk or preferential benefit-–perhaps baseline T-cell fitness, tumour microenvironment composition, or circulating tumour DNA dynamics. Fourth, dedicated studies in bendamustine-exposed and older populations are needed to guide patient selection.

For now, epcoritamab plus R² sets a new efficacy benchmark in relapsed follicular lymphoma. The challenge for clinicians will be translating this benefit to real-world patients while mitigating the accompanying toxicity burden-–a balance that will define whether this regimen becomes universally adopted or reserved for carefully selected candidates.
